# Aberrant neural correlates of multisensory processing of audiovisual social cues related to social anxiety: An electrophysiological study

**DOI:** 10.3389/fpsyt.2023.1020812

**Published:** 2023-01-24

**Authors:** Shuzhen Gan, Weijun Li

**Affiliations:** ^1^Shanghai Changning Mental Health Center, Shanghai, China; ^2^Shanghai Mental Health Center, Shanghai, China; ^3^Research Center of Brain and Cognitive Neuroscience, Liaoning Normal University, Dalian, China; ^4^Key Laboratory of Brain and Cognitive Neuroscience, Dalian, Liaoning, China

**Keywords:** social anxiety, multisensory processing, audiovisual integration, EEG, temporal dynamics, interpretation bias

## Abstract

**Background:**

Social anxiety disorder (SAD) is characterized by abnormal fear to social cues. Although unisensory processing to social stimuli associated with social anxiety (SA) has been well described, how multisensory processing relates to SA is still open to clarification. Using electroencephalography (EEG) measurement, we investigated the neural correlates of multisensory processing and related temporal dynamics in social anxiety disorder (SAD).

**Methods:**

Twenty-five SAD participants and 23 healthy control (HC) participants were presented with angry and neutral faces, voices and their combinations with congruent emotions and they completed an emotional categorization task.

**Results:**

We found that face-voice combinations facilitated auditory processing in multiple stages indicated by the acceleration of auditory N1 latency, attenuation of auditory N1 and P250 amplitudes, and decrease of theta power. In addition, bimodal inputs elicited cross-modal integrative activity which is indicated by the enhancement of visual P1, N170, and P3/LPP amplitudes and superadditive response of P1 and P3/LPP. More importantly, excessively greater integrative activity (at P3/LPP amplitude) was found in SAD participants, and this abnormal integrative activity in both early and late temporal stages was related to the larger interpretation bias of miscategorizing neutral face-voice combinations as angry.

**Conclusion:**

The study revealed that neural correlates of multisensory processing was aberrant in SAD and it was related to the interpretation bias to multimodal social cues in multiple processing stages. Our findings suggest that deficit in multisensory processing might be an important factor in the psychopathology of SA.

## 1. Introduction

Social anxiety Disorder (SAD) is mainly characterized by excessive fear of social interaction and the avoidance of social situations (1). Individuals with social anxiety (SA) typically differ from healthy controls in their perception of the outside world. They have hypervigilance and overreaction to social cues (2), which enhances fear and avoidance of social situations and bolsters the development of SA symptoms.

The cognitive model of SA proposes that a series of processing biases to social cues contribute to the symptoms (3). As faces and voices are two important sources of social evaluation, most studies have focused on them to reveal the cognitive and neural mechanisms of processing biases in SA. For instance, interpreting ambiguous or neutral faces and voices as negative was found in patients suffering from SAD and it is also considered as a key development and maintenance factor of SAD (4, 5). In addition, SAD patients and individuals who score high on SA assessments allocate more attention resources (6, 7), have more solid memorization (8, 9) to threatening faces and exhibit higher recognition accuracy for fearful and sad voices than their non-socially anxious counterparts (10). At the neural level, greater activity in response to faces and voices have been found in widely distributed networks at multiple temporal stages in SA individuals. For instance, the event-related potential (ERP) components of face processing, P1, N170, P3, and late positive potential (LPP), were enhanced in amplitude, which reflected the biases from early primary sensory processing to late sustained attention, semantic evaluation and maintenance to social cues (11). Spatially, the over responsivity of fusiform face areas (FFAs), temporal voice areas (TVAs), and limbic emotional areas (12–14) are associated with SA, as well as the aberrant functional connectivities of the emotional areas with sensory-specific areas and frontal regulatory areas (15, 16).

However, the majority of existing studies only revealed the unisensory visual or auditory processing bias since unimodal stimuli were used as experimental materials. In real life, social approval and rejection are usually conveyed *via* multiple modalities concurrently. A bulk of studies have demonstrated that emotional audiovisual inputs elicit multisensory processing, which facilitates emotion perception, including enhancing discrimination accuracy and shortening response time (17). Neuroimaging studies have showed that multisensory processing occurs in brain regions of different levels. Simultaneously presenting stimuli from the other modality enhances neural activity in sensory-specific cortices, such as FFA (18) and auditory-related anterior superior temporal sulcus (STS) (19). Contrary results were also found. Facilitated processing of decreased activity in response to bimodal audiovisual stimuli than that to unimodal ones was observed in the visual- (FFA, lateral occipital and posterior middle temporal gyrus) and auditory-specific cortices (anterior STS, middle STS) (20). In higher-order cortices, the bimodal inputs elicited integrative activity in heteromodal convergence regions such as posterior STS (21) and amygdala for emotional cues (22); and these supramodal cortices receive projections from the sensory-specific cortices for integrative processing (23). The electroencephalography (EEG) and magnetoencephalography (MEG) studies further revealed that multisensory processing modulates auditory and visual-sensitive ERPs and EEG oscillations. For instance, audiovisual inputs enhanced the amplitude of visual P1 and face-sensitive N170 (24, 25) and power of beta suppression (26) than the visual only condition. Contradictive findings, facilitated processing of visual N170, P200, and N300 components indicated by reduced latency and attenuated amplitude were also reported (20, 27). Meanwhile, auditory related N1 and P2 consistently exhibited facilitated processing in the presence of audiovisual stimuli compared to unimodal auditory ones (28, 29), as well as the power of auditory related theta and alpha oscillations (30). At the higher-order supramodal level, to identify the integrative activity from multisensory integrative neurons, the criterion of superadditivity was widely used in EEG studies, which was indicated by the larger activity in response to face-voice combinations (FV) than the sum of the activity in response to voices (V) and faces (F) [i.e., FV > (F + V)] (31). The superadditive response has been found in alpha, beta, and gamma oscillations (26, 32) and in ERPs from 40 to 400 ms after the stimuli onset (20, 33). In all, the findings from EEG studies indicated that the multisensory effect occurs at multiple temporal stages in wide frequency bands.

The neural basis of multisensory processing serves adaptive behavioral response through facilitating target identification and evaluation (34); whereas aberrant neural processing to multisensory inputs is related to various disorders, such as schizophrenia, depression, pervasive development disorder, and autism spectrum disorder (35–38). SAD is a typical disorder with impaired social perception and interaction, however, investigation on multisensory processing in SAD is still limited. One behavioral study reported that cross-modal facilitation from the bimodal face-voice inputs didn’t differ between people with high and low levels of SA (5). Kreifelts et al. (39) found that the middle STS had more integrative activity and greater functional connectivity with visual cortices in SAD patients compared to that in healthy controls; moreover, SAD patients had altered regions of maximal integrative activity within the STS. These findings indicated that although not revealed by behavioral metrics, altered function of multisensory processing was manifested at neural level in SAD.

The fMRI results revealed the spatial characteristic of the neural mechanism for its high spatial resolution, while the temporal dynamics of multisensory processing related to SA are still unknown. And EEG measurement can answer this question for its excellent temporal resolution. Second, cognitive bias is considered underlying the psychopathology (3) and a predictor of SA (40). Whether impaired multisensory processing contributes to the cognitive processing bias to social cues and influences the symptom severity of SA is open to clarification, and it would promote our understanding of the development and maintenance of SA in multimodal environment. Finally, fMRI studies usually applied a maximum criterion to identify integration {i.e., FV > [max (F, V)]}. Since the blood oxygen level-dependent BOLD response is derived from a heterogeneous population of neurons, the increased activity in response to bimodal inputs compared to that in response to unimodal ones identified in maximum criteria might be from the addition of the activity from separate auditory and visual processing, rather than from active integration (31). Applying the criterion of superadditivity instead would strongly point to active integration and help avoid this confounding. Here, we used EEG metrics to resolve these issues. To measure the multisensory processing, the cross-modal modulation of the face and voice-sensitive ERPs and EEG oscillations and cross-modal integration (i.e., superadditivity) of the bimodal-sensitive ERPs and EEG oscillations were investigated. SAD and healthy control (HC) participants were recruited. Angry and neutral faces, voices, and emotionally congruent face-voice combinations were presented to participants, and they were required to decide the emotional category of the stimuli. We predicted that SAD participants would differ from HC participants in both the amounts of cross-modal modulation of the voice- and face-sensitive indices and the cross-modal integration of bimodal-sensitive indices. In addition, we predicted that these neural indices of multisensory processing would be related to symptom severity as well as the interpretation bias to neutral bimodal face-voice combinations.

## 2. Materials and methods

### 2.1. Participants

Two hundred ninety-five college students from Liaoning Normal University were recruited through advertisements. They completed the Chinese version of Liebowitz SA Scale (LSAS) online to assess their level of social anxiety (41). According to previous studies (42), an LSAS score of 30 is the cutoff score to differentiate people with symptoms of SA from those without symptoms, and 60 is the cutoff score to differentiate people with generalized SA from those without generalized SA. People with generalized SA exhibit more broad impairments than those with non-generalized SA (43). Thus, participants with scores higher than 60 and those with scores lower than 30 were selected. Finally, 26 participants in the SAD group (6 male) and 24 in the HC group (10 male) were invited to complete the EEG experiment. In addition, all the selected participants underwent the Structured Clinical Interview for DSM-IV (SCID) by telephone. All of the participants in the SAD group met the criteria of DSM-IV of SAD, and two of them met the criteria of depression (mild to moderate). Both sex and age were counterbalanced between the two groups [sex: *χ^2^* = 1.98, *p* > 0.05; age: *t* (48) = 0.84, *p* > 0.05]. In the behavioral analysis, data from one participant in the SAD group were excluded due to low accuracy (lower than 50%). In the EEG analysis, data from one participant in the SAD group and one in the HC group were excluded due to high impedance (higher than 10 kΩ) and obvious and long-lasting drift, respectively. All participants were right-handed and reported normal or corrected-to-normal vision.

The exclusion criteria for both groups included a history of drug addiction, alcohol addiction, medication use within the last 2 weeks. All participants who completed the screening procedure were paid 10 yuan, and those who completed the EEG experiment were further paid 80 yuan. The Ethics Committee of Liaoning Normal University approved this study. Informed consent in accordance with the Declaration of Helsinki was signed by all participants.

### 2.2. Questionnaires

The LSAS is an extensively used questionnaire with 24 items measuring SA severity (41). Each item depicts a social situation that may evoke SA and people rated their feelings of anxiety (0, indicating none, to 3, indicating severe) and avoidance behaviors (0, indicating never, to 3, indicating usually) to the situation, separately. The range of the total score 0–144, with higher scores indicating more severe symptoms. The LSAS has been demonstrated to have a high internal consistency reliability above 0.90 in the Chinese population (44).

In addition to the LSAS, the Chinese version of the Social Avoidance and Distress Scale (SADS) was completed by participants after the EEG experiment (45). The SADS involves 28 items which are statements measuring aspects of social anxiety, including distress, discomfort, fear, anxiety, and the avoidance of social situations; participants had to decide whether each statement is true or false (46). The range of the total score 0–28, with higher scores indicating higher levels of avoidance and distress in social situation. The internal consistency reliability of the SADS is 0.85 in the Chinese population (47).

At last, the Chinese version of Beck Depression Inventory (BDI) was also completed by participants (45). The BDI measures the severity of depression with 21 items (48). Each item describes the severity of a mental or somatic symptom with four statements scored on a scale 0–3. The total score of BDI ranges from 0 to 63, with higher scores indicating more severe depression. And the BDI has high validity and reliability in Chinese population (49).

The participants’ characteristics of the two groups are displayed in Table 1.

**TABLE 1:** Mean and standard deviations (in parentheses) for the data of demographic measures and clinical measures of the SAD and HC groups.

	SAD (*N* = 26)	HC (*N* = 24)	*t* or χ^2^
Sex ratio (male/female)	6/20	10/14	*χ^2^* = 1.98
Age	21.77 (2.05)	22.38 (3.09)	*t* (48) = 0.84
LSAS	80.58 (11.82)	19.42 (5.98)	*t* (48) = 23.35***
SADS	18.12 (4.48)	5.38 (6.09)	*t* (48) = 8.47***
BDI	13.15 (9.23)	5.33 (3.33)	*t* (48) = 3.92***

LSAS, Liebowitz social anxiety scale; SADS, social avoidance and distress scale; BDI, beck depression inventory.

***p < 0.001.

### 2.3. Stimuli

Twenty college students from the Broadcasting and Hosting Art Department were recruited to record voice materials in a sound-proof room. They were instructed to articulate the interjection/Wei/in angry and neutral emotions. Each emotional voice was articulated twice. The durations of all the voices were edited to 350–370 ms with the software Praat (50), and the loudness was kept the same across angry and neutral emotions *via* the software Adobe Audition (Adobe Systems Inc., San Jose, CA, USA). Except for the duration and loudness, the voices were not changed in other parameters to maintain their naturality. Voices with poor quality after editing were excluded. Finally, 48 voices remained and were rated by another 25 college students. They were required to categorize the emotion of these voices as angry, neutral, happy, fearful, disgusted, sad or surprised. Then, emotional arousal was rated on a 9-point Likert scale (one, indicating no arousal at all or very calm, to nine, indicating extreme arousal, nervousness or excitement). According to the averaged accuracy of categorization for each voice, 16 angry and 16 neutral voices with the highest accuracies were selected, counterbalanced for sex (eight women and eight men in each emotion). The categorization accuracy and rating arousal are shown in Table 2. Angry and neutral faces, 16 for each emotion, were selected from the Chinese Facial Affective Picture System (CFAPS), which has been demonstrated to have a high reliability and identification rate in the portrayed emotions (51). Sex was counterbalanced in each emotion as well. Similar to the voices, these faces were also rated on their emotional category and arousal (see Table 2). The rating results suggested a higher arousal of angry stimuli than neutral stimuli [face: *t* (24) = 9.17, *p* < 0.0001; voice: *t* (24) = 10.43, *p* < 0.0001].

**TABLE 2:** Mean and standard deviations (in parentheses) for the categorization accuracy and rating arousal of faces and voices in each emotion.

	Face		Voice	
	Ang	Neu	Ang	Neu
Accuracy	0.61 (0.14)	0.79 (0.12)	0.63 (0.21)	0.85 (0.16)
Arousal rating	5.26 (1.28)	2.44 (1.46)	5.67 (1.28)	2.38 (1.69)

Ang, angry stimuli; Neu, neutral stimuli.

In addition, each face was paired with a voice with congruent emotion and the same sex, producing 32 face-voice combinations. In total, there were six types of stimuli, including angry faces, angry voices, angry bimodal combinations, neutral faces, neutral voices, and neural bimodal combinations. Each stimulus was presented twice in the experiment; thus, there were 192 stimuli in total.

### 2.4. Task design and procedure

In the experiment, the participants were presented with angry and neutral unimodal faces, voices, and bimodal combinations, and they had to decide the emotion of each stimulus. A mixed-design of group (SAD and HC) × modality (face, voice, and bimodal) × emotion (anger and neutrality) was used, with group as an intersubject factor and the other two components as intrasubject factors. The voices were presented binaurally with a pair of ear-hook headphones (Panasonic, RP-HS47GK). The loudness was adjusted to be comfortable for listening for each participant and was kept constant during the experiment. The faces were presented on the center of a black background screen with a visual angle of 40. The screen was 23 inches, with a refresh rate of 60 Hz.

After the participants signed informed consent, they were led into a dimly and sound-attenuated room and were comfortably seated in front of a computer monitor at a distance of 60–80 cm. Then, they were instructed to go through a practice procedure that involved 20 trials. After the participants became familiar with the task, they completed the experiment. The experiment lasted for 20–30 min and was divided into four sessions; participants could rest after completing each session. A total of 192 trials were included, with 32 trials for each condition. In a typical trial, a fixation would be presented for 500 ms, after which the stimuli would be displayed for 370 ms. After a black screen was displayed for 1,000 ms, a red dot appeared, and participants had to determine the emotion of the stimuli within 3,000 ms by pressing *f* or *j* on the keyboard with their left or right forefinger separately. Then the next trial began. The intertrial interval (ITI) jittered among 750, 1,000, and 1,250 ms, with a mean ITI of 1,000 ms. The key *f* indicated anger, whereas *j* indicated neutrality for half of the participants; keys were defined to indicate the opposite responses for the other half of the participants.

### 2.5. Electroencephalography data recording and processing

Continuous EEG data were recorded from 64 Ag/AgCI electrodes mounted in an elastic cap, which were placed based on the 10/20 system (ANT Neuro EEGO Inc., Germany). All the data collected with these electrodes were referenced online to CPz, amplified and digitized at a sampling rate of 500 Hz, and low-pass filtered at 100 Hz. The impedances of all electrodes were kept below 10.

The EEG data were preprocessed with EEGLAB (52) implemented in the MATLAB environment (The MathWorks Inc., Natick, MA, USA). The data were bandpass filtered at 1–30 Hz and rereferenced to the average of the left and right mastoids. The ocular artifacts were removed by independent component analysis (52). Then the continuous EEG data were segmented into epochs starting 200 ms before and ending 500 ms after stimulus onset, and prestimulus recording was used for baseline correction. Trials with excessive physiological artifacts exceeding ±100 μV were discarded. The percentages of trials left for each type of stimuli for the two groups were between 93 and 95%, with a grand average percentage of 94%. Then, the ERPs were obtained by averaging the trials within each condition in each electrode. For the time-frequency analysis, the epochs started 1,000 ms before stimulus onset and lasted for 2,000 ms. Then, the time-frequency power spectra were computed with the single-trial EEG data using a Hanning taper method with a sliding time window of 500 ms, ranging from 2 to 30 Hz in steps of 2 Hz and extending from −1,000 to 1,000 ms in steps of 20 ms. The longer duration for epoch than the windows of interest (−500–500 ms) was expected to prevent edge artifacts (53). Power spectra at each time-frequency point were averaged across trials within each condition and were normalized with the averaged power of the 500 ms prestimulus period with the decibel (dB) transform method [dB power = 10 × log_10_ (power/baseline)].

To identify the time windows and representative electrodes of ERP components sensitive to faces, voices, and bimodal combinations, we obtained grand-averaged peak latencies for these three types of stimuli in angry and neutral trials for SAD and HC participants separately according to their grand-averaged ERPs. Based on the topographies of the peak latencies, we constrained the electrodes for each component. This approach enabled our analysis to be independent from the expected difference and avoided circularity analysis (54). Accordingly, voices elicited a whole-scalp distributed N1 component with the maximum amplitudes in central electrodes (55) and a P250 component with a distribution in frontal and central electrodes (56) (see Figure 2B). Faces evoked a P1 component (57) and a N170 component both in the right occipital electrodes (58) and a P3/LPP component in parieto-occipital electrodes (59) (see Figure 4C). For bimodal combinations, a right occipitally distributed P1 component and a parieto-occipital P3/LPP component were observed (see Figure 5C). The time windows for these components are displayed in (Supplementary Table 1) and were all defined in windows around the grand-averaged peak latencies of the three types of stimuli; specifically, ±20 ms for auditory N1, ±40 ms for auditory P250, ±10 ms for visual P1 and ±20 ms for facial N170. For visual and bimodal evoked component P3/LPP, which had a positive deflection after 200 ms, the window was defined as 200–400 ms in all conditions (59). The amplitudes for these components were averaged within the corresponding time windows and electrodes.

**FIGURE 1:**
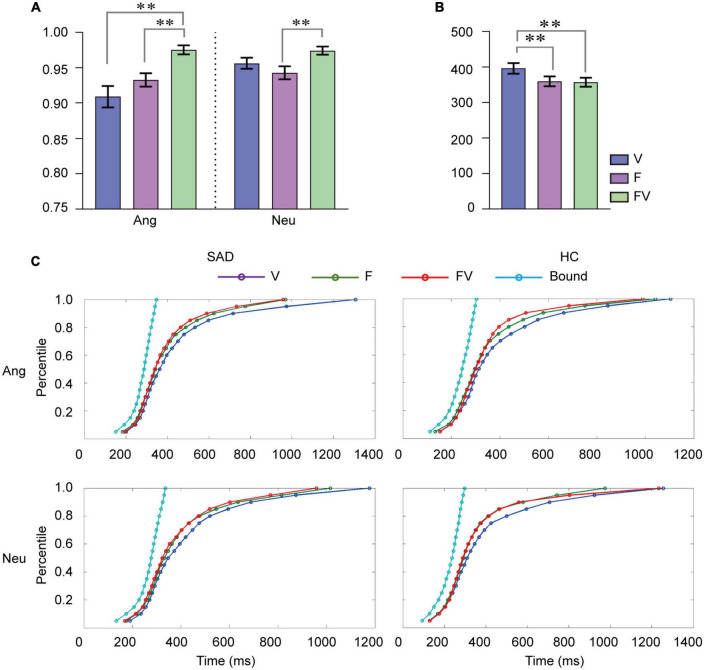
Behavioral results. **(A)** Mean categorization accuracy for faces, voices and bimodal combinations in angry, and neutral trials. **(B)** Mean RT (ms) to faces, voices and bimodal combinations. F, faces; V, voices; FV, bimodal combinations; Ang, angry trials; Neu, neutral trials. Error bars indicate ± SEM. **(C)** Cumulative density functions (CDFs) for F, V, and FV and the bound values predicted by RMI for RT in angry and neutral trials for the SAD and HC groups separately. ^**^p < 0.01.

**FIGURE 2:**
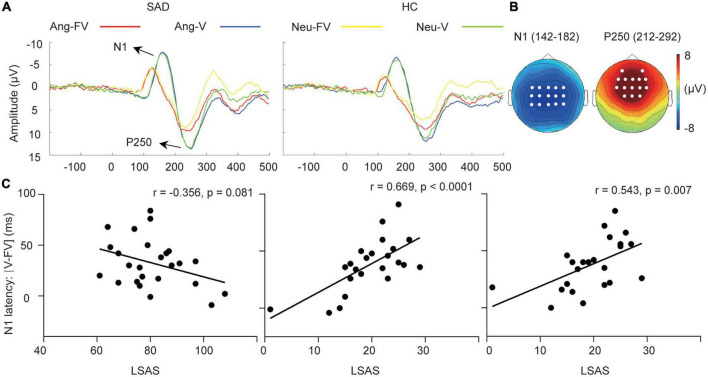
Cross-modal facilitation of auditory sensitive ERPs. **(A)** The grand-averaged waveforms of voices and bimodal combinations in angry and neutral trials for the SAD and HC groups. Ang-V, Ang-FV: Voices and bimodal combinations in angry trials separately; Neu-V, Neu-FV: Voices and bimodal combinations in neutral trials separately. **(B)** The topographies of N1 and P250 in their corresponding time windows (ms) elicited by neutral voices for the SAD group as an example. The white dots indicate the representative electrodes of the components. **(C)** The scatter plots of the LSAS scores and the facilitation of N1 latency (V-FV) in neutral trials for the SAD group (left) and in angry (middle) and neutral trials for the HC group (right).

**FIGURE 3:**
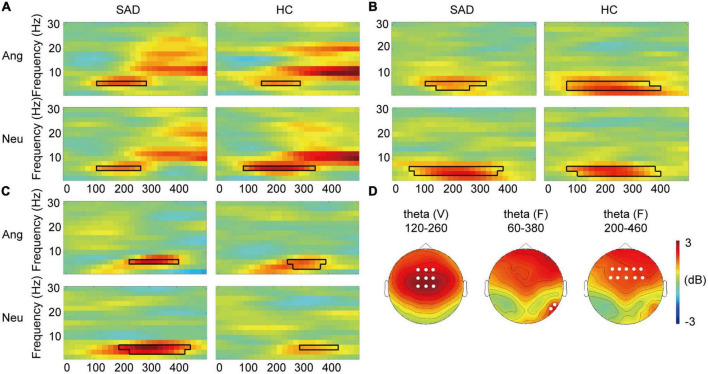
Cross-modal modulation of TFRs. **(A)** The grand-averaged TFRs of the difference between voices and bimodal combinations (V-FV) in angry and neutral trials for the SAD and HC groups across fronto-central electrodes. The boxes with black lines indicate the significant temporal-frequency clusters. **(B,C)** The grand-averaged TFRs of the difference between faces and bimodal combinations (FV-F) across occipital electrodes **(B)** and across frontal electrodes **(C)**. The boxes with black lines indicate the significant temporal-frequency clusters. **(D)** The topographies of voice-sensitive fronto-central theta (left), face-sensitive occipital theta (middle), and face- sensitive frontal theta (right) in neutral trials for the SAD group as examples. The white dots indicate the representative electrodes of the theta oscillations.

**FIGURE 4:**
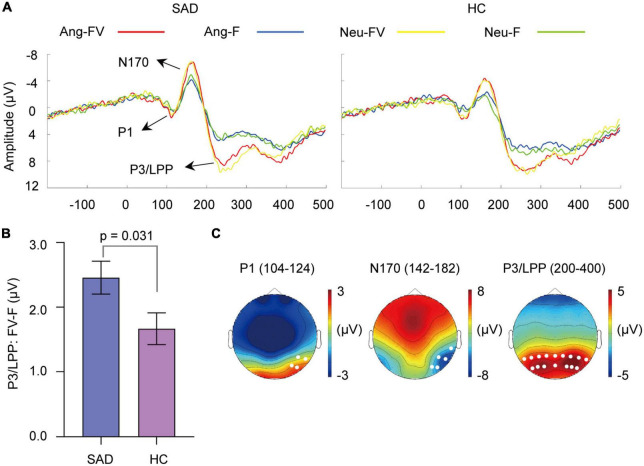
Cross-modal enhancement of visual sensitive ERPs. **(A)** The grand-averaged waveforms of faces and bimodal combinations in angry and neutral trials for the SAD and HC groups. Ang-F, Ang-FV: Faces and bimodal combinations in angry trials separately; Neu-F, Neu-FV: Faces and bimodal combinations in neutral trials separately. **(B)** The mean amount of enhancement of P3/LPP amplitude by bimodal combinations than faces (FV-F) for the SAD and HC groups. Error bars indicate ± SEM. **(C)** The topographies of P1, N170, and P3/LPP and their corresponding time windows (ms) elicited by neutral faces for the SAD group as an example. The white dots indicate the representative electrodes of the components.

**FIGURE 5:**
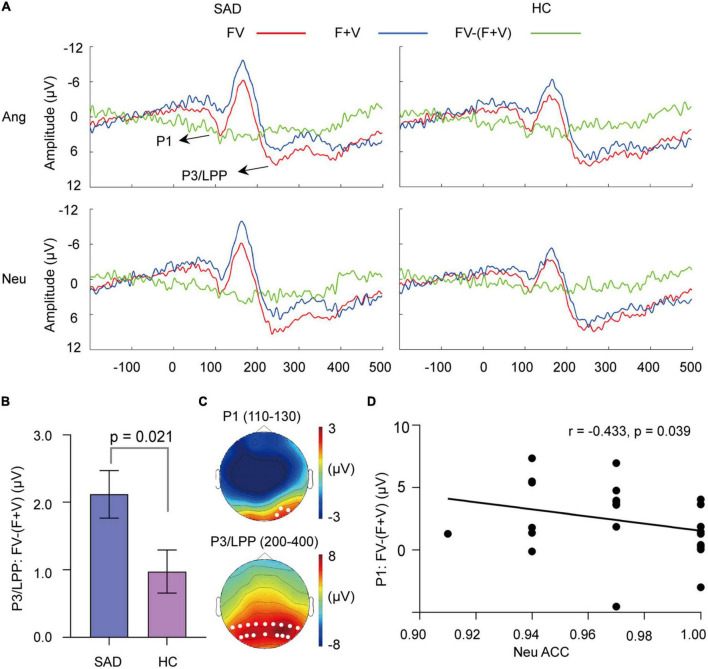
The superadditivity of ERPs. **(A)** The grand-averaged waveforms under the FV and (F + V) conditions and their difference waves [FV–(F + V)]. **(B)** The mean values of [FV–(F + V)] on P3/LPP amplitude for the SAD and HC groups. Error bars indicate ± SEM. **(C)** The topographies of P1 and P3/LPP under FV condition for the SAD group in neutral trials as an example. The white dots indicate the representative electrodes of the components. **(D)** The scatter plot of the categorization accuracy to neutral FV stimuli and values of [FV–(F + V)] on the P1 amplitude in neutral trials for the SAD group.

For the time-frequency data, we focused theta (4–6 Hz) event-related synchronization (ERS) and alpha (8–12 Hz) event-related desynchronization (ERD). Similar to the ERP components, the representative electrodes for each frequency band were predefined according to the visual inspection of the scalp distribution of the grand-averaged time-frequency representation (TFR) for each of the three types of stimuli to avoid circularity analysis. The averaged TFR showed theta ERS in right occipital electrodes (60) and frontal electrodes (61) and alpha ERD with maximal modulation in parieto-occipital electrodes for faces (62). For voices, the averaged TFR showed theta ERS with maximal power in fronto-central electrodes (63). For the bimodal combinations, theta ERS in right occipital electrodes and frontal electrodes and alpha ERD in parieto-occipital electrodes were observed. The topographies of the ERS/ERD and their representative electrodes are indicated in Figure 3D, and the power for each frequency point was averaged across these electrodes. As no effect was found in further statistical analysis for the cross-modal modulation effect in face related alpha ERD and the superadditivity effect in bimodal related theta ERS and alpha ERD, their topographies of activity were not presented.

### 2.6. Statistical analyses

To investigate the multisensory processing in behavioral data, the categorization accuracy (percent correct response) and reaction times (RTs) were first subjected to a group (SAD and HC) × modality (F, V, and FV) × emotion (anger and neutrality) analysis of variance (ANOVA). Then, the increase in accuracy for bimodal combinations compared to that for unimodal faces (FV-F) and voices (FV-V), and the decrease of RT to bimodal combinations compared to that to faces (F-FV) and voices (V-FV) for each subject were calculated to indicate the behavioral gain from bimodal inputs than unimodal ones. Next, a group × emotion ANOVA was performed on these difference values to examine whether the amount of behavioral gain was different between the groups. In addition, a procedure of Miller’s Race Model Inequality (RMI) (64) was followed for each emotion and group separately to examine whether the acceleration of RT to bimodal combinations was from a statistical facilitation (“race” between the processes of the two modalities) or from cross-modal interaction. In the RMI, the cumulative density functions (CDFs) of RT for faces, voices, bimodal combinations, and the predicted values of the race model (i.e., the sum of the CDF of faces and voices) which indicate a statistical facilitation from bimodal inputs, were calculated at every 5th percentile, ranging from 0.05 to 1.0. Then, the predictions were compared with the RTs to bimodal combinations with a paired *t*-test at each percentile. The violation of the race model was indicated by a longer RT to bimodal combinations at a timepoint than the predicted values.

To investigate the multisensory effect in ERP data, two levels of statistical analysis were performed. First, we examined how bimodal inputs modulate unisensory ERPs and EEG oscillations. For the auditory indices, group (SAD and HC) × modality (V and FV) × emotion (anger and neutrality) ANOVAs were performed on the voice-sensitive N1 and P250 amplitudes. In addition, the grand average waves showed a difference in the peak latencies of N1; thus, the N1 latencies to voices and bimodal combinations for each subject were obtained and evaluated by the same test. For the visual indices, group (SAD and HC) × modality (F and FV) × emotion (anger and neutrality) ANOVAs were performed on the amplitudes of face-sensitive P1, N170, and P3/LPP. Furthermore, whether the amount of cross-modal modulation differed between SAD and HC was examined. The difference values between the activity of bimodal and unimodal conditions were calculated and subjected to a repeated-measures ANOVA with the factors group and emotion.

In the second level of analysis for multisensory processing, we examined the integrative activity to bimodal inputs, which was identified by superadditivity, the larger amplitude elicited by bimodal combinations (FV) than the sum of the amplitudes elicited by faces and voices (F + V). A group (SAD and HC) × modality [FV and (F + V)] × emotion (anger and neutrality) ANOVA was conducted on the amplitudes of P1 and P3/LPP. To investigate whether the superadditive response differed between the groups, the difference values calculated by [FV−(F + V)] were subjected to the ANOVA with the factors group and emotion. In addition to the traditional component analysis, a data-driven approach was used to investigate the spatiotemporal characteristic of superadditivity. The comparisons of FV and (F + V) conditions were performed in each electrode-time point in angry and neutral trials in the SAD and HC groups separately. To control type I errors from multiple comparisons, the Monte–Carlo non-parametric cluster-based permutation test implemented in Fieldtrip (65) was used. This procedure involved merging the data of the FV and (F + V) conditions and randomly partitioning them into two sets as two pseudo-conditions of modality. Then, a *t*-test was performed in each electrode-time point in the window 0–500 ms. Adjacent samples exceeding a significant alpha level of 0.01 were grouped into a cluster with a minimum of two neighboring electrodes. Then, the *t*-values of the points within each cluster were summed as the cluster-level statistic. With 2,000 iteration of randomly partitioning FV and (F + V) conditions, a null distribution of cluster-level statistics was established. If the cluster-level statistic calculated from the actual FV and (F + V) conditions was larger than the 95% percentile of the null distribution, the difference between the two conditions was considered significant. Furthermore, we examined the group difference in the superadditive response. The values [FV−(F + V)] were calculated and entered into comparisons of the SAD and HC groups in angry and neutral trials separately. A similar cluster-based permutation test was applied to identify the significant spatiotemporal clusters.

For the time-frequency data, a statistical procedure similar to that used for the ERP data was applied. The non-parametric cluster-based permutation test was used to identify the temporal-frequency clusters of the modulation effect on face and voice-sensitive ERS and ERD and the superadditivity on bimodal stimuli-elicited spectral perturbations. Then, the group difference was also examined for the cross-modal facilitation of unisensory-sensitive ERS/ERD and integrative responses of bimodal sensitive ERS/ERD.

In addition to the group difference, we investigated how multisensory processing related to the severity of SA and categorization bias in angry and neutral trials within the SAD and HC groups separately by Pearson correlation tests. First, correlations between the LSAS scores and the accuracy and RTs in response to face-voice combinations were performed to check whether SA was related to a categorization bias to bimodal social stimuli in our study. Then, how the amount of cross-modal modulation of unisensory ERPs and EEG oscillations and the superadditive response related to LSAS scores and behavioral responses (accuracy, RTs) to bimodal inputs were examined. As depression is highly comorbid with social anxiety (66), we calculated the partial correlations with BDI scores controlled when the BDI scores were significantly correlated to the behavioral indices, the amount of cross-modal modulation and superadditive response.

Finally, we examined whether SAD participants differed from HC participants on unisensory ERP and EEG responses. ANOVAs with the factors group and emotion were conducted on unimodal voice-evoked and face-evoked ERP data separately. Non-parametric cluster-based permutation tests were applied to compare the group difference on the voice and face-evoked ERS/ERD in angry and neutral trials separately.

Analyses for ERP components and Pearson correlations were performed using SPSS version 20.0 (IBM, Armonk, NY). Greenhouse–Geisser corrections were applied when the sphericity hypothesis was violated. Bonferroni corrections were also used in multiple comparisons.

## 3. Results

### 3.1. Behavioral results

Participants responded to stimuli from different modalities with different accuracies [*F*_(2,94)_ = 16.20, *p* < 0.001, $\eta_{p}^{2}$= 0.256]. Accuracy was also higher in angry trials than in neutral ones [*F*_(1,47)_ = 5.918, *p* < 0.05, $\eta_{p}^{2}$ = 0.112]. There was a significant interaction between modality and emotion [*F*_(2,94)_ = 6.960, *p* < 0.01, $\eta_{p}^{2}$ = 0.129]. Simple effect analysis revealed that the modality effect for both angry and neutral stimuli was prominent [anger: *F*_(2,94)_ = 15.46, *p* < 0.0001; neutrality: *F*_(2,94)_ = 5.61, *p* < 0.01]. Participants responded to bimodal combinations more accurately than both voices [*t* (48) = 5.131, *p* < 0.001] and faces [*t* (48) = 5.386, *p* < 0.001] in angry trials. In neutral trials, we observed an increase in accuracy for bimodal combinations compared with faces [*t* (48) = 3.633, *p* < 0.001], while a difference between bimodal combinations and voices was not observed (*p* > 0.05) (see Figure 1A). No effect of group or its interaction with modality and emotion was found (*ps* > 0.05).

The analyses on RT indicated that the main effect of modality was significant [*F*_(2,94)_ = 10.888, *p* < 0.001, $\eta_{p}^{2}$ = 0.188), and participants responded to voices more slowly than to faces [*t* (48) = 3.364, *p* < 0.01] and bimodal combinations [*t* (48) = 4.666, *p* < 0.001] (see Figure 1B). No additional main effect or interactions was revealed (*ps* > 0.05). In addition, the RMI test indicated no shorter RT to bimodal inputs than that indicated by the model prediction at any percentile in either angry or neutral trials in either group, which suggested that the RT data did not show any facilitation effect from multisensory processing (see Figure 1C).

The behavioral gain in accuracy and RT did not reveal any effect of group difference, emotion, or their interaction (*ps* > 0.05).

### 3.2. Cross-modal modulation of auditory ERPs and EEG oscillations

#### 3.2.1. N1 component

Shorter latency [*F*_(1,46)_ = 134.576, *p* < 0.0001, $\eta_{p}^{2}$ = 0.745] and smaller amplitude of N1 [*F*_(1,46)_ = 44.623, *p* < 0.0001, $\eta_{p}^{2}$ = 0.492] were elicited by bimodal inputs compared with that elicited by voices (see Figure 2A). In addition, SAD participants showed a larger amplitude compared with HC participants [*F*_(1,46)_ = 5.23, *p* = 0.027, $\eta_{p}^{2}$ = 0.102]. The emotion effect was not found (*p* > 0.05).

The analysis of the reduced latency (V-FV) and decreased amplitude (FV-V) of N1 did not reveal any effect of group or emotion (*ps* > 0.05).

#### 3.2.2. P250 component

Decreased amplitude of P250 was observed in response to bimodal inputs than that to voices [*F*_(1,46)_ = 17.682, *p* < 0.0001, $\eta_{p}^{2}$ = 0.278] (see Figure 2A). Angry trials had larger amplitudes than neutral trials [*F*_(1,46)_ = 15.978, *p* < 0.0001, $\eta_{p}^{2}$ = 0.258). An interaction between modality and emotion was found (*F*_(1,46)_ = 9.721, *p* = 0.003, $\eta_{p}^{2}$ = 0.174). Simple effect analysis revealed that bimodal combinations had a lower amplitude than voices in both angry [*F*_(1,46)_ = 9.40, *p* = 0.004] and neutral trials [*F*_(1,46)_ = 27.09, *p* < 0.0001]. The group effect was not observed, either its interactions with modality and emotion (*ps* > 0.05).

The analysis of the amplitude decrease (V-FV) of P250 revealed a significant effect of emotion [*F*_(1,46)_ = 9.721, *p* = 0.003. *η_*p*_^2^* = 0.174], with angry trials having a larger amplitude decrease than neutral trials. We found no group difference in the attenuation of amplitude (*p* > 0.05).

#### 3.2.3. Electroencephalography oscillations

In the SAD group, the TFR results showed a decreased power in frontal theta ERS in response to bimodal combinations compared to that to voices during the window of 120–280 ms in angry trials (*T*_*sum*_ = 33.741, *p* = 0.004) and the window of 120–260 ms in neutral trials (*T*_*sum*_ = 28.551, *p* = 0.010) at 6 Hz. In the HC group, this decrease was observed in a cluster during 160–280 ms in angry trials (*T*_*sum*_ = 26.615, *p* = 0.008) and a cluster during 100–340 ms in neutral trials (*T*_*sum*_ = 75.034, *p* < 0.0005) at 6 Hz (see Figure 3A). We found no group difference in the power decrease (V-FV) in theta band (*p* > 0.05).

### 3.3. Cross-modal modulation of visual ERPs and EEG oscillations

#### 3.3.1. P1 component

The amplitude of P1 was enhanced by bimodal inputs than that elicited by faces [*F*_(1,46)_ = 11.685, *p* = 0.001, $\eta_{p}^{2}$ = 0.203] (see Figure 4A). The effect of group, emotion or their interaction with modality was not observed (*ps* > 0.05).

The analysis of the amplitude enhancement (FV-F) of P1 did not reveal any significant effect of group or emotion (*ps* > 0.05).

#### 3.3.2. N170 component

Increased amplitude of N170 was found in response to bimodal inputs than to faces [*F*_(1,46)_ = 39.913, *p* < 0.0001, $\eta_{p}^{2}$ = 0.465] (see Figure 4A). The SAD group showed a trend of increasing amplitude compared with the HC group [*F*_(1,46)_ = 3.174, *p* = 0.081, $\eta_{p}^{2}$ = 0.065]. The effect of emotion or the interactions among the factors was not found (*ps* > 0.05).

The increase in N170 amplitude (F-FV) did not differ between the two groups or between the angry and neutral trials (*ps* > 0.05).

#### 3.3.3. P3/LPP component

Enhanced P3/LPP amplitude was observed in response to bimodal inputs than to faces [*F*_(1,46)_ = 135.397, *p* < 0.0001, $\eta_{p}^{2}$ = 0.746] (see Figure 4A). The group effect was not significant (*p* > 0.05), but it interacted with modality [*F*_(1,46)_ = 4.959, *p* = 0.031, $\eta_{p}^{2}$ = 0.097]. Simple analysis indicated that amplitude enhancement was found in both the SAD [*F*_(1,46)_ = 100.27, *p* < 0.0001] and HC groups [*F*_(1,46)_ = 42.49, *p* < 0.0001]. The emotion effect was not found (*p* > 0.05), but its interaction with modality was prominent [*F*_(1,46)_ = 5.135, *p* = 0.028, $\eta_{p}^{2}$ = 0.100], with the bimodal combinations eliciting a larger amplitude than faces in both angry [*F*_(1,46)_ = 129.41, *p* < 0.0001] and neutral [*F*_(1,46)_ = 66.86, *p* < 0.0001] trials.

The enhancement of P3/LPP amplitude (FV-F) was greater in the SAD group than in the HC group [*F*_(1,46)_ = 4.959, *p* = 0.031, $\eta_{p}^{2}$ = 0.097] (see Figure 4B) and greater in angry trials than in neutral trials [*F*_(1,46)_ = 5.135, *p* = 0.028, $\eta_{p}^{2}$ = 0.100].

#### 3.3.4. Electroencephalography oscillations

In the SAD group, the power of occipital theta was increased in response to bimodal combinations compared with that to faces in both angry (*T*_*sum*_ = 56.799, *p* = 0.003) and neutral trials (*T*_*sum*_ = 158.591, *p* < 0.0005), with a window of 120–320 ms and 60–380 ms, respectively, at 4–6 Hz. This enhancement in the HC group was found in the cluster during 60–400 ms in angry trials (*T*_*sum*_ = 149.632, *p* < 0.0005) and in the cluster during 80–400 ms in neutral trials (*T*_*sum*_ = 163.065, *p* = 0.0005) at 4–6 Hz (see Figure 3B). In addition to the occipital theta, bimodal combinations increased the power of frontal theta compared with that elicited by faces in 260–400 ms at 6 Hz in angry trials (*T*_*sum*_ = 30.783, *p* = 0.004) and in 200–460 ms at 4–6 Hz in neutral trials (*T*_*sum*_ = 94.653, *p* < 0.0005) in the SAD group. In the HC group, the effect was observed in 260–380 ms at 4–6 Hz in angry trials (*T*_*sum*_ = 40.617, *p* = 0.0015) and in 300–420 ms at 6 Hz in neutral trials (*T*_*sum*_ = 22.299, *p* = 0.007) (see Figure 3C). The alpha ERD data did not differ between bimodal face-voice combinations and unimodal faces (*p* > 0.05).

The analysis of the power enhancement (FV-F) of occipital theta or frontal theta did not reveal any cluster for group difference (*ps* > 0.05).

### 3.4. Superadditivity of bimodal-sensitive ERPs and EEG oscillations

#### 3.4.1. P1 component

Superadditivity was found [*F*_(1,46)_ = 70.184, *p* < 0.0001, $\eta_{p}^{2}$ = 0.604], with bimodal FV having a larger P1 amplitude than (F + V) condition (see Figure 5A). The emotion effect was not observed (*p* > 0.05), but its interaction with modality was found [*F*_(1,46)_ = 6.006, *p* = 0.018, $\eta_{p}^{2}$ = 0.115]. Simple analysis indicated that superadditivity was significant in both angry [*F*_(1, 46)_ = 71.27, *p* < 0.001] and neutral trials [*F*_(1,46)_ = 27.18, *p* < 0.001]. The group effect was not observed, either its interaction with modality and emotion (*ps* > 0.05).

The analysis of superadditive response [FV−(F + V)] indicated that the angry trials had greater superadditive response than neutral trials [*F*_(1,46)_ = 6.006, *p* = 0.018, $\eta_{p}^{2}$ = 0.115]. No group difference was observed (*p* > 0.05).

#### 3.4.2. P3/LPP component

Face-voice elicited larger amplitude of P3/LPP than (F + V) condition [*F*_(1,46)_ = 41.969, *p* < 0.0001, $\eta_{p}^{2}$ = 0.477] (see Figure 5A). An interaction between modality and group was observed [*F*_(1,46)_ = 5.742, *p* = 0.021, $\eta_{p}^{2}$ = 0.111], with superadditivity being prominent in both the SAD [*F*_(1,46)_ = 41.09, *p* < 0.001] and HC groups [*F*_(1, 46)_ = 8.00, *p* = 0.007].

The analysis of [FV−(F + V)] revealed a significant group effect [*F*_(1,46)_ = 5.742, *p* = 0.021, $\eta_{p}^{2}$ = 0.111], with SAD participants having a greater superadditive response than HC participants (see Figure 5B). Neither emotion effect nor its interaction with group was revealed (*ps* > 0.05).

#### 3.4.3. Event-related potential clusters

**FIGURE 6:**
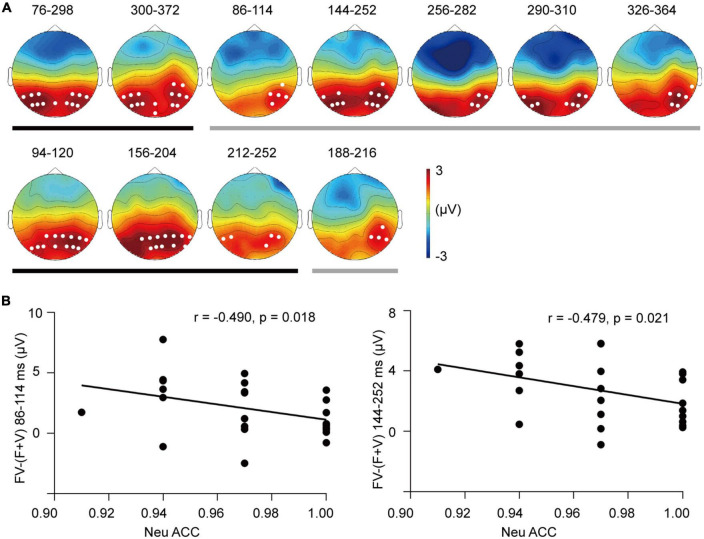
The superadditivity of ERP clusters. **(A)** The topographies and their corresponding time windows (ms) for the clusters of superadditivity for the SAD (up row) and HC groups (bottom row). Black line: Angry trials; gray line: Neutral trials. **(B)** Scatter plots of the categorization accuracy in response to neutral bimodal combinations and superadditive response [FV–(F + V)] in the clusters during 86–114 ms (left) and 144–252 ms (right) for the SAD group.

In the permutation test, superadditivity was distributed in parieto-occipital electrodes in each emotion and group (see Figure 6A). In the SAD group, angry FV elicited a more positive ERP than (F + V) condition in two clusters, with one in 76–298 ms (*T*_*sum*_ = 9326.535, *p* < 0.001) and the second in 300–372 ms (*T*_*sum*_ = 2551.240, *p* = 0.002). Superadditivity in neutral trials was found in five clusters of dispersive windows, with cluster 1 in 86–114 ms (*T*_*sum*_ = 420.253, *p* = 0.045), cluster 2 in 144–252 ms (*T*_*sum*_ = 4075.514, *p* < 0.0001), cluster 3 in 256–282 ms (*T*_*sum*_ = 595.878, *p* = 0.025), cluster 4 in 290–310 ms (*T*_*sum*_ = 666.1098, *p* = 0.022), and cluster 5 in 326–364 ms (*T*_*sum*_ = 852.303, *p* = 0.015). In the HC group, superadditivity was observed in three clusters in angry trials, with cluster 1 in 94–120 ms (*T*_*sum*_ = 1090.467, *p* = 0.012), cluster 2 in 156–204 ms (*T*_*sum*_ = 1976.955, *p* = 0.003), and cluster 3 in 212–252 ms (*T*_*sum*_ = 716.432, *p* = 0.027). A marginally significant cluster was found in neutral trials in 188–216 ms (*T*_*sum*_ = 344.235, *p* = 0.052).

The analysis of superadditive response [FV−(F + V)] did not reveal any group difference in either angry or neutral trials (*ps* > 0.05).

#### 3.4.4. Electroencephalography oscillations

No superadditivity was found in either theta ERS or alpha ERD, and no group difference was found in the superadditive response (*ps* > 0.05).

### 3.5. Correlation results

The symptom severity was correlated with the categorization in response to neutral bimodal combinations in the SAD group (*r* = −0.428, *p* = 0.037), and participants with higher LSAS scores had lower categorization accuracy in response to neutral FV. In the HC group, this correlation was not found (*p* > 0.05).

For the cross-modal facilitation of unimodal-sensitive indices, a marginally significant correlation was found between the LSAS scores and the acceleration of N1 latency in neutral trials in the SAD group (*r* = −0.356, *p* = 0.081), suggesting that SAD participants with more severe symptoms tended to have smaller facilitation from bimodal inputs on auditory processing. In the HC group, in contrast, higher LSAS scores were related to greater acceleration of N1 latency in both angry (*r* = 0.669, *p* < 0.0001) and neutral trials (*r* = 0.543, *p* = 0.007) (see Figure 2C). No correlation was observed for the visual indices (*ps* > 0.05).

For the integrative activity, we found that greater superadditive response [FV−(F + V)] of P1 amplitude was associated with lower accuracy of categorizing neutral FV stimuli in the SAD group (*r* = −0.433, *p* = 0.039) (see Figure 5D). In addition, the superadditive response was also negatively related to the categorization accuracies to neutral FV stimuli in the clusters of 86–114 ms (*r* = −0.490, *p* = 0.018) and 144–252 ms (*r* = −0.479, *p* = 0.021) in the SAD group (see Figure 6B), with participants who had a greater superadditive response having more errors in categorizing neutral bimodal combinations.

### 3.6. Unimodal stimulus-sensitive EEG analysis

#### 3.6.1. Visual-evoked ERPs and EEG oscillations

The group effect on N170 amplitude was found to be marginally significant [*F*_(1,46)_ = 3.28, *p* = 0.077, $\eta_{p}^{2}$ = 0.067], with the SAD group having larger amplitudes than the HC group. The emotion effect was not found (*p* > 0.05). The interaction between group and emotion was significant [*F*_(1,46)_ = 4.413, *p* = 0.041, $\eta_{p}^{2}$ = 0.088], with more negative N170 in the SAD than HC group in neutral trials [*F*_(1,46)_ = 4.55, *p* = 0.038], and this difference was not observed in angry trials (*p* > 0.05). None of the group difference was found in other visual-evoked ERP components P1, P3/LPP, theta ERS, or alpha ERD (*ps* > 0.05).

#### 3.6.2. Auditory-evoked ERPs and EEG oscillations

For the N1 amplitude, neither the group nor the emotion effect was prominent (*ps* > 0.05), and the interaction between them was not observed (*p* > 0.05). Planned simple effect analysis indicated that the SAD group tended to have larger amplitudes than the HC group in neutral trials [*F*_(1,46)_ = 3.50, *p* = 0.068] but not in angry trials (*p* > 0.05). None of the group or emotion effect was found on the N1 latency, P250 amplitude, or power of theta ERS (*ps* > 0.05).

## 4. Discussion

Our real life is full of information from multiple modalities, and social interaction relies on perceiving and conveying auditory and visual information concurrently. Thus, the integration of multisensory input is a critical component for precepting the outside world. Previous studies have widely revealed that the neural correlates of biased unisensory processing was underlying the psychopathology of SA, however, whether the aberrant neural correlates of multisensory processing were related to SA and its temporal dynamics are still open to clarification. With EEG metrics, the current study revealed that multisensory processing had aberrant neural correlates in socially anxious individuals; and in multiple temporal stages, it was related to the interpretation bias to neutral audiovisual social cues of SA.

In the behavioral results, we observed that all participants had higher accuracy and shorter RTs when categorizing the emotion of the bimodal face-voice combinations than when categorizing unimodal ones, which suggested that audiovisual inputs promoted the identification of emotion (17). The RMI test on RTs did not reveal any violation of the model as expected. A possible reason for this outcome is that the RTs in our study measured the delayed reaction but not the immediate reaction. Alternatively, the emotional intensity of the voices and faces used in our study were strong enough and easy to be identified, and the cross-modal interaction measured by the RMI test did not occur. Based on the principle of inverse effectiveness, when the signals from the different modalities are weaker and more ambiguous, a larger performance benefit was gained from their combinations; otherwise, the benefit from cross-modal interaction would decrease or disappear due to a ceiling effect (67). In addition, the group difference in behavioral gain was not found, which is consistent with a previous study (5). This might suggest that the behavioral metrics were not sensitive to individual differences in multisensory processing.

For the auditory sensitive components, the latency of central N1 was reduced, and its amplitude as well as fronto-central P250 amplitude were decreased when faces were simultaneously presented compared with that when single voices were presented (26, 28). As central N1 and fronto-central P250 indicate early detection (68, 69) and classification to auditory stimuli (55), respectively, the facilitation of their latency and amplitude might reflect faster early detection and increased processing efficiency to the auditory part in bimodal combinations. Consistent with the ERP finding, the time-frequency indicator fronto-central theta also had decreased power to bimodal combinations with similar temporal dynamics (around 100–340 ms) and supported the attenuated processing effort (30, 70). These cross-modal facilitation effects on auditory processing were consistent with previous studies and might reflect a top-down cross-modal prediction (28, 71, 72). In our study, since the face images were presented instantaneously and the voices were unfolding temporally, face processing would be faster than the voice processing. Therefore, some features of voices, such as emotion and sex, can be predicted by faces which would make voice processing easier and faster.

The cross-modal facilitation of auditory activity tended to be more impaired in individuals with more severe symptoms, indicated by the marginally significant correlation of the higher LSAS scores and smaller facilitation of N1 latency in neutral trials in the SAD group. The impaired facilitation might be related with the lack of cognitive resources in SAD participants. Since cross-modal prediction is a top-down process and relies on available resources (73), whereas the limited resources in individuals with SAD were usually occupied by their worried thoughts (74), thus the effect of cross-modal prediction was reduced in those with more severe symptoms. In angry trials, we didn’t observe the reduced cross-modal facilitation accompanied with the increased SA level, and this result might be related to the bias of negative anticipation in SAD. When angry voice appears, it is congruent with the participants’ anticipation and its processing was facilitated. In the HC group, contradict result was found, those with higher level of SA had greater facilitation of auditory processing. HC participants had intact and sufficient cognitive resources left for cross-modal prediction and their low-to-mild range of anxiety might play a positive role in voice processing. It had been demonstrated that mild anxiety improves motivation and makes participants more focused on the target information and accelerates processing (75); thus, we observed that the acceleration of N1 latency in both angry and neutral trials was greater along with the higher level of SA.

Compared to the attenuation of the auditory ERPs, the visual-sensitive components, right occipital P1, N170 and parieto-occipital P3/LPP were all had enhanced amplitudes in response to face-voice combinations (24, 25). Due to our design of the simultaneous onset of faces and voices, the facial processing cannot be cross-modally predicted by voices. As suggested by previous studies, the larger EEG activity in visual-sensitive brain regions in response to audiovisual inputs than unimodal visual ones possibly reflect integrative processing (76). And our ERP results further revealed that the integrative activity existed in multiple temporal stages, i.e., early attention-boosted general visual processing (56), face-specific processing (57), and later semantic evaluation and maintenance (58, 77). Consistently, theta ERS showed increased power to bimodal inputs in a broad time window (78), i.e., the visual-related occipital theta power (around 60–400 ms) which was linked to early sensory processing to late sustained attention (79, 80), and the late frontal theta power (around 200–500 ms) which reflecting higher-order processes (81, 82).

The enhanced visual EEG responses to audiovisual inputs reflecting cross-modal integration were further confirmed by the superadditivity of right occipital P1 and parieto-occipital P3/LPP elicited by bimodal inputs, which had similar topography distribution and temporal dynamics as the visual-sensitive components. The cluster-based permutation tests replicated the effect of superadditivity found in component analysis with similar posterior topography, and this topography was also consistent with previous studies using data-driven analysis (33). However, prior studies also displayed the cross-modal integration occurred in fronto-central electrodes (83). This discrepancy might arise from the different ERP generators related to integration. As found by previous studies, multisensory integrative neurons are distributed widely in visual, auditory and supramodal cortices (84). Further MEG studies might help to elucidate the generator of superadditivity of these posterior-distributed ERPs.

More importantly, the integrative activity to bimodal inputs showed a group difference which was indicated by the late component P3/LPP. This finding suggested that SAD had excessive neural activity of cross-modal integration specifically in a late stage, which might contribute to abnormal high-level processes. The results of the permutation test provided convergent evidence on the temporal dynamics of abnormal integration related to SA, in which the superadditivity in SAD participants was sustained until 360–370 ms, while in HC participants, it ended approximately 200–250 ms after stimulus onset. Previous studies demonstrated that socially anxious people had sustained attention allocation, long-lasting dwelling time and more solid memory to unimodal social cues (11, 85), whereas our finding suggested that this processing bias also existed in integrating social cues from different modalities. In addition to the general severity of symptoms, cross-modal integration was associated with a key cognitive factor that contributed to the symptom development and maintenance, i.e., the bias of interpretating neutral or ambiguous social cues as threatening (86). In cluster analysis, larger superadditive responses [FV−(F + V)] in parietal and occipital electrodes in both early (86–114 ms) and late (144–252 ms) windows were associated with participants’ increased propensity to categorize neutral face-voice combinations as angry ones. And the component analysis confirmed this correlation in occipital P1. It has been demonstrated that integrative activity serves the behavioral gain of emotion categorization (34), whereas our results further indicated that the aberrant integrative activity from the early to late stages contributes to the interpretation bias in SA.

The findings promoted the understanding of the psychopathology of SA in daily multimodal situations and the relationship between multisensory processing and psychiatric disorders. Previous studies have suggested that patients hyporeactive to social cues usually have reduced integrative activity, such as that observed in pervasive development disorder (37) and autism spectrum disorder (87). Our results suggested that hyperactivation in response to social interaction might be related to excessive integrative activity. The over activity of integration might increase the perceived emotional salience of neutral bimodal social cues through the projection from the integrative regions of STS (88) to the amygdala, hence contributing to a typical cognitive manifestation of SA, overestimating the threat of bimodal social cues and miscategorization of neutral or blurred information, and that further contribute to the development and maintenance of the symptoms.

Several limitations of our study should be noted. The static facial expressions were paired with voices and used as bimodal combinations, which limited the ecological validity of the study. Dynamic videos of facial expressions would synchronize better with voices and constitute more natural stimuli. Second, we used the criteria of superadditivity to measure the integration of inputs from auditory and visual modalities at neural level. However, one of the challenges of using this criterion is common activation (31). When processing all three types of visual, auditory and bimodal inputs, neural activity such as motor and anticipatory activity that is not related to auditory or visual processing is also involved. When calculating superadditive response [FV−(F + V)], this activity was subtracted twice. To resolve this flaw, the null stimulus (N) condition should be included to subtract the irrelevant activity from each condition {i.e., [(FV + N)−(F + V)]} (31). Third, the sex ratio was not strictly matched across the two groups. A supplementary analysis revealed that the unmatched sex ratio between the two groups didn’t influence the group effect on neural indices of multisensory processing (see Supplementary material). However, the small number of males and females in the two groups might prevent drawing a stable conclusion. Finally, a larger sample size in future work would be helpful for providing more reliable results for the correlation analysis.

Overall, the present study revealed that the neural correlates of multisensory processing was aberrant in SAD and it was related to interpretation bias to bimodal social cues in multiple processing stages in SAD individuals. First, SAD participants had excessive larger integrative activity in a late processing stage measured by the enhancement and superadditive response of parieto-occipital P3/LPP. Second, the over-integrative activity from the early to the late stage was associated with the negative interpretation bias to neutral bimodal stimuli in SAD participants. These results suggested that aberrant neural correlates of multisensory processing might be an important component in psychopathology of SA.

## Data availability statement

The raw data supporting the conclusions of this article will be made available by the authors, without undue reservation.

## Ethics statement

The studies involving human participants were reviewed and approved by the Ethics Committee of Liaoning Normal University. The patients/participants provided their written informed consent to participate in this study.

## Author contributions

SG carried out literature research, designed the experiment, analyzed the data, and wrote and revised the manuscript. WL collected data, revised the manuscript, and approved the final version. Both authors contributed to the article and approved the submitted version.
