# Supplementary material for: Aberrant neural correlates of multisensory processing of audiovisual social cues related to social anxiety: An electrophysiological study
Source: Front Psychiatry. 2023 Jan 24;14:1020812. doi: 10.3389/fpsyt.2023.1020812 (PMC9902659; doi:10.3389/fpsyt.2023.1020812)
Supplement: Supplementary file 1 [file Data_Sheet_1.docx]

Supplementary Material

# sTable 1. The time windows for each component.

| tests | component |  |  | time window (ms) |
| --- | --- | --- | --- | --- |
| V vs FV | N1 | SAD | AngV | 142-182 |
|  |  |  | AngFV | 110-150 |
|  |  |  | NeuV | 142-182 |
|  |  |  | NeuFV | 108-148 |
|  |  | HC | AngV | 144-184 |
|  |  |  | AngFV | 106-146 |
|  |  |  | NeuV | 148-188 |
|  |  |  | NeuFV | 110-150 |
|  | P250 | SAD | AngV, FV | 212-292 |
|  |  |  | NeuV, FV | 212-292 |
|  |  | HC | AngV, FV | 216-296 |
|  |  |  | NeuV, FV | 212-292 |
| F vs FV | P1 | SAD | AngF, FV | 108-128 |
|  |  |  | NeuF, FV | 104-124 |
|  |  | HC | AngF, FV | 104-124 |
|  |  |  | NeuF, FV | 102-122 |
|  | N170 | SAD | AngF, FV | 142-182 |
|  |  |  | NeuF, FV | 142-182 |
|  |  | HC | AngF, FV | 144-184 |
|  |  |  | NeuF, FV | 142-182 |
|  | P3/LPP | SAD, HC | All conditions | 200-400 |
| FV vs F+V | |  |  |  |
|  | P1 | SAD | Ang FV, [F+V] | 102-122 |
|  |  |  | Neu FV, [F+V] | 110-130 |
|  |  | HC | Ang FV, [F+V] | 104-124 |
|  |  |  | Neu FV, [F+V] | 102-122 |
|  | P3/LPP | SAD, HC | All conditions | 200-400 |

V, voices; F, faces; FV, face-voice combinations; Ang, angry trials; Neu, neutral trials.

# Supplementary analysis of examining the influence of sex ration on group effect on neural indices of multisensory processing

To investigate whether the sex ratio influences the group effect (SAD vs. HC) on neural indices of multisensory processing, we included the sex as an independent variable into the ANOVAs to examine whether it interacted with group effect. As the group differences were found in two ERP indices in our former analysis, i.e., the cross-modal enhancement on the amplitude of visual-sensitive P3/LPP (i.e., [FV-F]) and the superadditive response of bimodal elicited P3/LPP (i.e., [FV-(F+V)]), the sex (male, female) × group (SAD, HC) × emotion (anger, neutrality) repeated-measures ANOVAs were conducted in these two indices.

For the cross-modal enhanced amplitude of visual sensitive P3/LPP (i.e., [FV-F]), the effect of sex was not found (*F*(1,44) = 0.079, *p* = 0.78) and it didn’t interact with group (*F*(1,44) = 0.715, *p* = 0.402) or any other factors (*ps* > 0.05). The group effect was observed (*F* (1,44) = 5.17, *p* = 0.028, $\eta_{p}^{2}$ = 0.105), with the SAD group having larger cross-modal enhancement on P3/LPP amplitude than the HC group.

For the superadditive response of bimodal elicited P3/LPP (i.e., [FV-(F+V)]), the effect of sex was still not significant (*F* (1,44) = 1.412, *p* = 0.241), as well as its interaction with group (*F* (1, 44) = 0.054, *p* = 0.817) or other factors (*ps* > 0.05). The group effect was stably observed (*F* (1,44) = 4.323, *p* =0.043, $\eta_{p}^{2}$ = 0.089), with the SAD group having larger superadditive response than the HC group.

These results suggested that the group differences in neural indices of multisensory processing was not influenced by the unmatched sex ratios in the SAD and HC groups.
